# Color memory as a diagnostic test for mild cognitive impairment and early stage of Alzheimer’s disease

**DOI:** 10.3389/fneur.2025.1589335

**Published:** 2025-05-27

**Authors:** Vaiva Sutnikiene, Egle Audronyte, Gyte Pakulaite-Kazliene, Gintaras Kaubrys

**Affiliations:** Clinic of Neurology and Neurosurgery, Institute of Clinical Medicine, Faculty of Medicine, Vilnius University, Vilnius, Lithuania

**Keywords:** Alzheimer’s disease, color memory, color recognition memory, color vision, mild cognitive impairment

## Abstract

**Background:**

Color vision and memory are potential markers of Alzheimer’s disease (AD); however, information regarding their changes in early AD remains limited.

**Objective:**

The aim of this study was to evaluate color recognition memory in mild cognitive impairment (MCI), early Alzheimer’s disease (AD), and older adults with normal cognition, as well as to assess its diagnostic properties.

**Methods:**

We included 27 participants with mild dementia (MD), 25 with MCI, and 25 older adults with normal cognition who served as the control group (CG). Participants completed the Mini-Mental Status Examination (MMSE), the Clinical Dementia Rating (CDR), and the Alzheimer’s disease Assessment Scale-Cognitive Subscale (ADAS-Cog 13) for cognitive assessment; delayed word recall (after 30 min), the Ishihara test, and a color recognition memory test were also administered. The color recognition memory test was performed using a basic 12-color kit displayed on a computer screen. Color recognition was evaluated after 5 and 30 min using four initial stimuli and four new colors from the 12-color kit. Participants were required to recognize which of the eight randomly presented colors they had previously seen.

**Results:**

Significant differences were observed in the total error scores for color recognition memory among the three groups. Medians (interquartile range) for CG, MCI, and MD groups were 2 (2), 4 (2), and 5 (3) respectively. A Kruskal–Wallis test returned a significant *p*-value (*p* < 0.001); *post-hoc* analysis of group pairs was then conducted for CG and MCI (*p* < 0.001), MCI and MD (*p* = 0.007), and CG and MD (*p* < 0.001). Using demographic information and ADAS-Cog 13 scores as predictors, a multinomial logistic regression model accurately categorized 77.9% of cases (96% of CG, 64% of MCI, and 74.1% of MD cases). Adding the color memory total error score to the model improved accuracy to 84.4% (100% of CG, 76% of MCI, and 77.8% of MD cases).

**Conclusion:**

Color recognition memory test results differed significantly between participants with normal cognition and those with MCI and early AD. Therefore, it may help in the early diagnosis of AD as a simple, non-invasive diagnostic marker.

## 1 Introduction

Alzheimer’s disease (AD) is the most common cause of dementia, accounting for 60–80% of cases, and causes progressive neurodegeneration and cognitive decline (1, 2). According to data from the Framingham Heart Study, the lifetime risk of Alzheimer’s dementia at age 45 is 20% for women and 10% for men, indicating that 1 in 5 women and 1 in 10 men of this age could eventually develop AD (3). Nonetheless, according to estimates from the Alzheimer’s Disease International, approximately 75% of individuals with dementia worldwide remain undiagnosed, and as many as 85% do not receive post-diagnostic support services (4).

Recent discoveries of disease-modifying treatments have been associated with a growing need to develop tools for detection of AD at the earliest clinically determinable stage. However, an audit of memory services in England revealed that less than 2% of patients underwent molecular confirmation of their disease using cerebrospinal fluid biomarkers (5). Moreover, the 2024 Alzheimer’s disease International report found that over 90% of caregivers and respondents from the public were more inclined to seek a diagnosis if a treatment that could modify the course of the disease was accessible (6). The lack of a valid and reliable cognitive test with minimal influence from language differences which is comparable across multinational and multilingual populations is a concern, and using digital language-neutral cognitive evaluations is encouraged (4).

Visual alterations emerge early in the progression of AD and may serve as potential noninvasive markers of AD-related neurodegeneration (7). AD-related neuropathological lesions have been identified throughout the visual system, including the retina, resulting in various signs and symptoms (7–9). Visual issues reported in AD include abnormalities in contrast sensitivity, visual field deficits, abnormalities in eye movements related to fixation and saccades, and abnormalities in visual functions which lead to impaired reading ability, challenges in recognizing objects and shapes, and locating items in one’s environment (7–11). Furthermore, the progression from normal cognitive function to mild cognitive impairment (MCI) is linked to decreased visual acuity, suggesting that the impact of vision impairment is greatest during the early stages of cognitive decline (12). According to recent studies, worsening contrast sensitivity over time is associated with a higher likelihood of incident dementia (13). Visual recognition memory impairment also occurs early in the course of MCI (14). One of the 10 signs of AD in the 2024 Alzheimer’s Association Report was difficulty in understanding visual images and spatial relationships. As a result, patients with AD experience difficulties in estimating distances and perceiving colors and contrasts, which can lead to challenges while driving (2). Color vision and memory are potential markers as well; however, these visual functions are not usually analyzed. Thus, information about their association with the degree of cognitive impairment remains limited.

Cholinergic loss is one of the most prominent components of AD neuropathology (15). The nucleus basalis of Meynert contains acetylcholine-producing neurons which are essential for regulating various aspects of visual perception (16). In the visual perception pathway, the ventral stream, which involves interactions among visual areas V1, V2, and V4, plays a crucial role in recognizing and identifying shapes and colors. This pathway projects to regions of the inferior and medial temporal lobe, the latter of which is responsible for encoding long-term memories (17). Therefore, impairment of color perception and memory may be promising as potential non-invasive functional markers of early AD.

Recent studies provide growing evidence that color perception can aid in distinguishing AD from other forms of dementia (18–20). Usually, only one aspect of visual perception is analyzed, such as color vision or color discrimination, with limited information regarding color memory, color recognition, and long-term memory retention in AD. This could be because color vision and memory assessments are challenging to incorporate into clinical practice due to the complexity of evaluating responses, and the tools required. Using the Ishihara test, Arnaoutoglou et al. concluded that color perception differentiates AD from vascular dementia (18). The color recognition tasks require information about the color hue that was seen, and its comparison with the hue that was presented. This leads to widespread cortical activation (21). The impairment of cortical activation may represent early changes in AD (15, 20).

This study aimed to examine color recognition memory in patients with early AD and cognitively normal older adults, as well as to evaluate its potential as a diagnostic tool. We hypothesized that the ability to recognize colors is compromised in MCI and early AD, which could effectively distinguish participants with AD from those who were cognitively healthy.

## 2 Materials and methods

### 2.1 Participants

The study included three groups; 27 subjects diagnosed with mild dementia due to Alzheimer’s disease (MD), 25 subjects with amnestic mild cognitive impairment (MCI), and 25 cognitively healthy older adults who served as the control group (CG).

Participants diagnosed with MD met the criteria for probable AD as outlined by the National Institute on Aging and Alzheimer’s Association (NIA/AA) (22) and scored 1 on the Clinical Dementia Rating Total Score (CDR-TS). Subjects in the MCI group met the NIA/AA criteria for clinical MCI attributed to AD (23), and scored a CDR-TS of 0.5. Cognitively healthy older adults in the CG exhibited no cognitive complaints, scored 0 on the CDR-TS and displayed no signs of neurological disorders.

Participants were recruited from the Memory Clinic at Vilnius University Hospital, Santaros Klinikos. Using established criteria, a specialist in cognitive neurology determined a probable AD diagnosis, which was validated by the observed progression of cognitive deterioration indicative of an ongoing pathological process (22). For a subset of patients with AD (6/27, or 22%), medical records contained information about positive AD cerebrospinal fluid (CSF) biomarker status, which confirmed the AD diagnosis according to the 2018 NIA-AA (National Institute on Aging and Alzheimer’s Association) (NIA-AA) research framework (24). MCI due to AD was identified by implementing clinical and cognitive criteria, and the underlying cause of MCI was found to align with the AD pathophysiological process (23). This diagnostic approach involved excluding vascular, traumatic, and medical factors contributing to cognitive decline and documenting progressive cognitive deterioration over time (23). Biomarker analysis suggested a moderate probability that participants’ MCI was linked to AD, as structural magnetic resonance imaging (MRI) exhibited indications of neuronal damage. For a subset of patients with MCI (7/25, or 28%), medical records contained information about positive AD CSF biomarker status, confirming the AD diagnosis (24). Due to the exploratory nature of our study, CSF biomarker analysis was not performed within the framework of this study.

This study excluded individuals with neurological conditions affecting the central nervous system (except for MCI and MD); cerebrovascular disorders (indicated by a Hachinski Ischemic Score of 4 or higher); prior head injury; major psychiatric conditions (e.g., schizophrenia, delirium, psychosis, or depression with a Geriatric Depression Scale score exceeding 9); vision and hearing disorders impairing cognitive testing; known color vision impairment from a young age; significant ophthalmologic conditions (glaucoma, cataract, diabetic retinopathy, age-related macular degeneration); diagnosed or symptomatic significant cardiovascular, hepatic, or metabolic disorders; substance abuse; and use of psychotropic drugs. The study adhered to the 1975 Helsinki Declaration guidelines and was approved by the Vilnius Regional Bioethics Committee (Approval Number 2022/1-1405-877). All study participants provided informed consent in writing prior to their involvement in the research.

### 2.2 Assessments of cognitive function and color vision

The Mini-Mental State Examination (MMSE) was used to assess overall cognitive function. For a more comprehensive cognitive evaluation, participants completed the 13-item version of the Alzheimer’s disease Assessment Scale-Cognitive Subscale (ADAS-Cog 13). This assessment encompasses various cognitive domains, including word recall after 5 minutes, commands, constructional praxis, delayed word recall, naming, ideational praxis, orientation, word recognition, remembering test instructions, comprehension of spoken language, word-finding difficulty, spoken language ability, and number cancelation. The ADAS-Cog 13 employs a scoring system ranging from 0 to 85, with higher scores indicating impaired cognitive function. The Clinical Dementia Rating Sum of Boxes (CDR-SB) scale was used to assess the extent of cognitive decline and functional impairment. Color vision was assessed using a paper-based version of the Ishihara test. Plates 1–15 from the 2021 edition of the 24-plate Ishihara test were presented and the number of errors was recorded. According to the test instructions, plates 1–15 determine the normality of color vision. Color vision was considered normal if 13 or more plates were read correctly (two or fewer errors).

### 2.3 Assessment of color recognition memory

Color recognition memory was evaluated using a 12-color kit displayed on a computer screen. The personal computer monitor parameters were set to factory default. The computer monitor was warmed for at least 30 min and the target luminance value was 120 cd/m^2^ (for a moderately lit room). Monitor parameters were checked using simple visual/eye-based monitor calibration images from Lagom LCD test, and a color recognition task was developed using the Microsoft PowerPoint. The color memory recognition task was divided into three parts: stimulus presentation and delayed recognition after 5 and 30 min. Four initial stimulus colors were randomly selected and presented on a grid background for 5 s each, with an interstimulus interval of 5 s to reduce the impact of the color’s after-effect. Participants were instructed to memorize the colors since they would be asked to identify the ones that they had seen. Hues were selected using the RGB (*red-green-blue*) color model, which is commonly used to represent and display images on electronic systems (such as computers). In this model, the red, green, and blue colors are combined in various proportions to produce other colors. Each pair of colors in the RGB scale differed by at least 64 units in the red, green, or blue range (0–255). The 12 colors used were categorized using basic color names: red, green, blue, yellow, orange, brown, pink, violet, black, white, gray, and light blue (separate categorical names in some languages like Lithuanian, Greek, Italian, and Russian). The RGB values were as follows: red (255, 0, 0), green (0, 255, 0), blue (0, 0, 255), yellow (255, 255, 0), orange (255, 128, 0), brown (128, 64, 0), pink (255, 125, 192), violet (192, 0, 255), light blue (128, 192, 255), black (0, 0, 0), white (255, 255, 255), and gray (128, 128, 128). The participants were not asked to name the colors, but to memorize them for later identification.

In the delayed recognition task, 5 min after stimulus presentation, participants were required to recognize which of the eight randomly presented colors (four initial stimulus colors and four randomly selected colors from the 12-color kit) they had previously seen.

**Figure 1:**
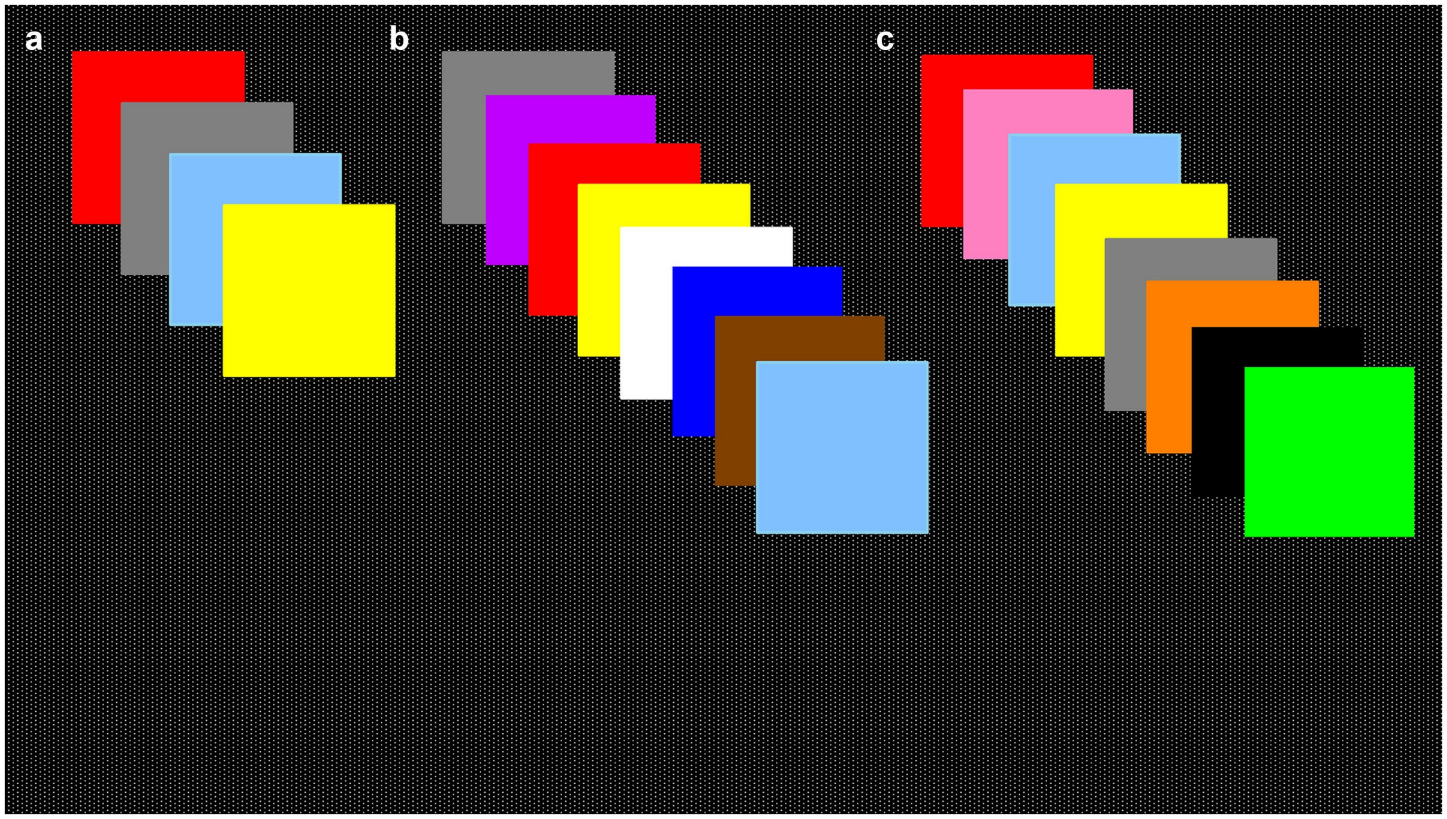
Color recognition memory task, stimuli colors. **(a)** Four initial stimulus colors were randomly selected and displayed on the computer screen for 5 s, each with an inter-stimulus interval of 5 s. Color memory was evaluated by color recognition after 5 and 30 min using the four initial stimuli and four new colors from kits **(b,c)**.

After 30 min, participants were asked to identify which of the eight presented colors they had previously seen (four initial stimulus colors and four new colors from the 12-color kit that had not been used in the previous delayed recognition task) (Figure 1).

### 2.4 Data analysis

Statistical analysis was performed using the IBM SPSS Statistics version 26.0. To establish adequate study power, we calculated the required number of subjects using G_Power for Windows version 3.1. Power (1-beta) was set at 0.85, the level of significance at the level of *p* < 0.05, one-way, and the effect size was set at 0.4, as expected to find large effect sizes (f). The Kruskal–Wallis test required a minimum of 72 participants. The Shapiro–Wilk test was used to assess data normality. Differences in demographic and clinical data, as well as test results among groups, were examined using Kruskal–Wallis or chi-square tests. In addition, verbal memory performance was assessed by the total medians (IQR) of the error score of words not recalled out of 10 after 5 and 30 min between these groups. Furthermore, the color recognition error scores medians (IQR) after 5 and 30 min for the CG, MCI, and MD were evaluated. Correlations of the total error scores for color memory with cognitive test results and demographic data were calculated using Spearman’s rank correlation coefficient. Multiple linear regression models adjusted for age, education, sex, Ishihara test, and cognitive test scores (MMSE, ADAS-Cog-13, and CDR-SB) as independent variables were used to determine whether these factors could significantly predict the total error scores observed in the color memory tasks. The diagnostic characteristics of color recognition memory were assessed and compared with those of verbal memory (based on the ADAS-Cog 13 delayed word recall after 5 and 30 min). The cut-off scores for the total error score of the color memory task were established as indicative of pathology (MCI and MD), MCI, and MD. To examine the association between the independent variables and their classification into three groups, a multinomial logistic regression analysis was conducted. Initially, a model was developed using age, education, sex, and ADAS-Cog 13 score as predictor variables. Second, word recall after 30 min was incorporated into the model. Finally, color recognition after 30 min was added to the initial model. The fit of the model to the data while adding predictor variables and the classification accuracies of the models were calculated. A *p*-value of 0.05 was set at a baseline of statistical significance and adjusted for multiple comparisons using Bonferroni correction.

## 3 Results

### 3.1 Demographic and clinical characteristics

The distribution of sexes was comparable across all three groups, with no statistically significant differences observed (chi-square test, *p* > 0.05). Similarly, the Kruskal–Wallis test revealed no significant differences in educational background, age, Geriatric Depression Scale scores (indicating depressive symptoms), or Hachinski ischemic scores among the groups (*p* > 0.05). The demographic and clinical characteristics of the patients are presented in Table 1.

**Table 1:** Demographic and clinical characteristics of the participants.

	CG (*N* = 25)	MCI (*N* = 25)	MD (*N* = 27)	Statistics (*χ*^2^(2)/H(2), *p*)
Male (%) *	13 (52%)	7 (28%)	10 (37%)	3.09, 0.21
Years of education *	16 (2)	15 (4)	15 (3)	4.06, 0.132
Age *	73 (9)	75 (11)	77 (9)	4.39, 0.111
GDS *	4 (2)	4 (1.5)	4 (2)	1.58, 0.453
HIS *	1 (1)	1 (1)	1 (1)	2.04, 0.36

Unless otherwise indicated, data are expressed as the median along with the interquartile range. * Groups did not differ significantly. MD, mild dementia; MCI, mild cognitive impairment; CG, control group; GDS, Geriatric Depression Scale; HIS, Hachinski Ischemic Score.

The Kruskal–Wallis test indicated significant differences in cognitive tests performance across the three groups (*p* < 0.05), as confirmed by the *post-hoc* analysis.

The medians (interquartile ranges [IQR]) of the Ishihara test results (number of errors in 15 plates) for the CG, MCI, and MD groups were 0 (0), 0 (1), 1 (3) respectively; the Kruskal–Wallis test indicated a significant difference (H[2] = 12.27, *p* = 0.002). *Post-hoc* analysis also showed significant differences between MD and CG (*p* < 0.001). However, no significant differences were observed between the CG and MCI, or the MCI and MD groups (*p* > 0.05). Detailed results of the cognitive assessment and color vision are presented in Table 2.

**Table 2:** Cognitive tests and color vision results of the participants.

	CG (*N* = 25)	MCI (*N* = 25)	MD (*N* = 27)	Statistics (H(2), *p*)
MMSE *	29 (1)	25 (2)	22 (2)	68.45, <0.001
ADAS-Cog 13 *	12 (5.5)	22 (7.66)	31 (7.67)	51.4, <0.001
CDR Sum of Boxes *	0 (0)	1.5 (0.8)	4.5 (0.5)	70.63, <0.001
Ishihara test **	0 (0)	0 (1)	1 (3)	12.27, 0.002

The data are expressed as the median along with the interquartile range. * There were significant differences among the three groups. ** There was significant difference only between MD and CG. MD, mild dementia; MCI, mild cognitive impairment; CG, control group.

Verbal memory performance was assessed by the number of words not recalled out of 10 after 5 and 30 min. The total medians (IQR) of the error score of words not recalled for the CG, MCI, and MD groups are 9 (3), 15 (5), and 16.67 (2.165) respectively. The Kruskal–Wallis test indicated a statistically significant difference (H[2] = 36.18, *p* < 0.001). *Post hoc* analysis revealed significant differences among the three groups (*p* < 0.001). Medians (IQR) of verbal memory errors after 5 min for the three groups were 4 (1.34) for CG, 6 (1.67) for MCI, and 7 (1.5) for MD. The Kruskal–Wallis test indicated a statistically significant difference (H[2] = 29.32, *p* < 0.001). *Post hoc* analysis revealed significant differences among the three groups (*p* < 0.001). Medians (IQR) of verbal memory errors after 30 min were 5 (2), 9 (4), and 10 (0) for the CG, MCI, and MD groups, respectively. The Kruskal–Wallis test indicated a statistically significant difference (H[2] = 36.13, *p* < 0.001). Post hoc analysis revealed significant differences among the three groups (*p* < 0.001).

### 3.2 Color recognition memory

Medians (IQR) of the color recognition total error scores for CG, MCI and MD were 2 (2), 4 (2), and 5 (3) respectively; the Kruskal–Wallis test indicated a significant difference (H[2] = 34.36, *p* < 0.001). *Post-hoc* analysis revealed statistically significant differences between CG and MCI (*p* < 0.001), MCI and MD (*p* = 0.007), and CG and MD (*p* < 0.001). Total error score for each color in three groups is provided in Supplementary Table 1.

Color memory task scores after 5 min were evaluated across the three groups, and the medians (IQR) were found to be 0 (2), 1 (2), and 3 (2) for CG, MCI, and MD respectively; Kruskal–Wallis H(2) = 31.11, *p* < 0.001; *post-hoc* examination revealed statistically significant differences between the groups: CG vs. MCI, *p* = 0.002; MCI vs. MD, *p* = 0.002; CG vs. MD, *p* < 0.001.

Color memory task scores after 30 min were evaluated across the three groups, and the medians (IQR) for CG, MCI and MD were 1 (2), 2 (2), and 3 (1) respectively; Kruskal–Wallis H(2) = 34.36, *p* < 0.001; *post-hoc* analysis revealed significant differences between the two groups: CG vs. MCI, *p* = 0.001; CG vs. MD, *p* < 0.001, but no significant differences were observed between MCI and MD, *p* = 0.15.

Among all participants, the total error scores for color memory were strongly correlated with the MMSE results (Spearman’s rho: −0.647; *p* < 0.001) and moderately correlated with CDR-SB (Spearman’s rho: 0.593; *p* < 0.001), ADAS-Cog 13 (Spearman’s rho: 0.568; *p* < 0.001), and verbal memory (Spearman’s rho: 0.492; *p* < 0.001).

When examining separate groups, no notable associations were found between the performance on color memory tasks and cognitive test scores, participant age, or education.

The multiple linear regression showed statistical significance in the adjusted model with MMSE (*R*^2^ = 0.378, *F* = 10.23, *β* = −0.668, *p* < 0.001), ADAS-Cog 13 (*R*^2^ = 0.274, *F* = 6.74, *β* = 0.588, *p* < 0.001), and CDR-SB (*R*^2^ = 0.324, *F* = 8.3, *β* = 0.616, *p* < 0.001).

In all of the models, color memory scores were not significantly influenced by other variables such as age, sex, education level, and Ishihara test results (*p* > 0.05).

### 3.3 Diagnostic characteristics of color recognition memory in comparison with verbal memory

**Figure 2:**
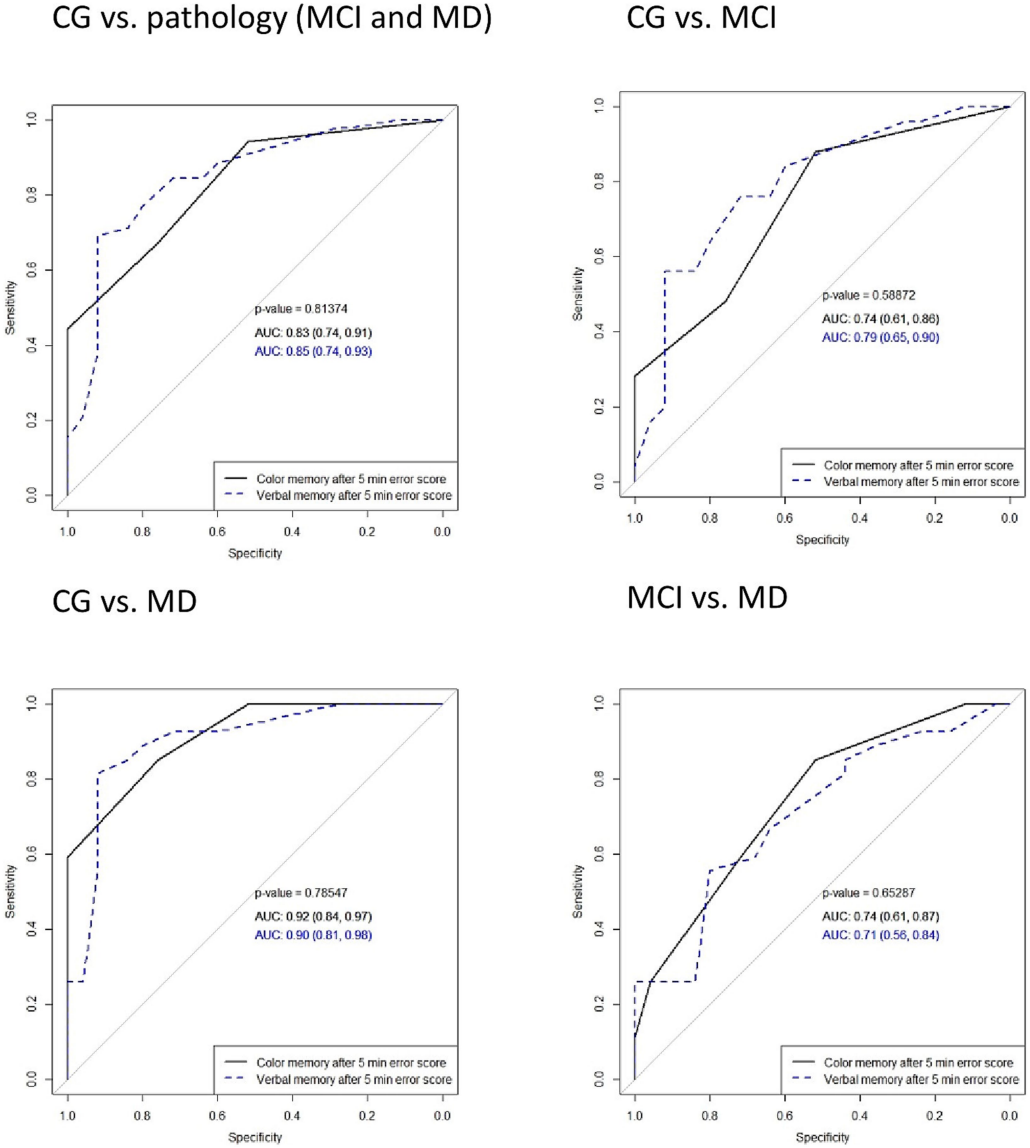
Comparisons of areas under the ROC curves (AUC) in delayed word recall and color recognition after 5 min in verbal and color memory tasks in distinguishing between participant groups. Solid lines represent color memory and dashed lines represent verbal memory test results. MD, mild dementia; MCI, mild cognitive impairment; CG, control group.

**Figure 3:**
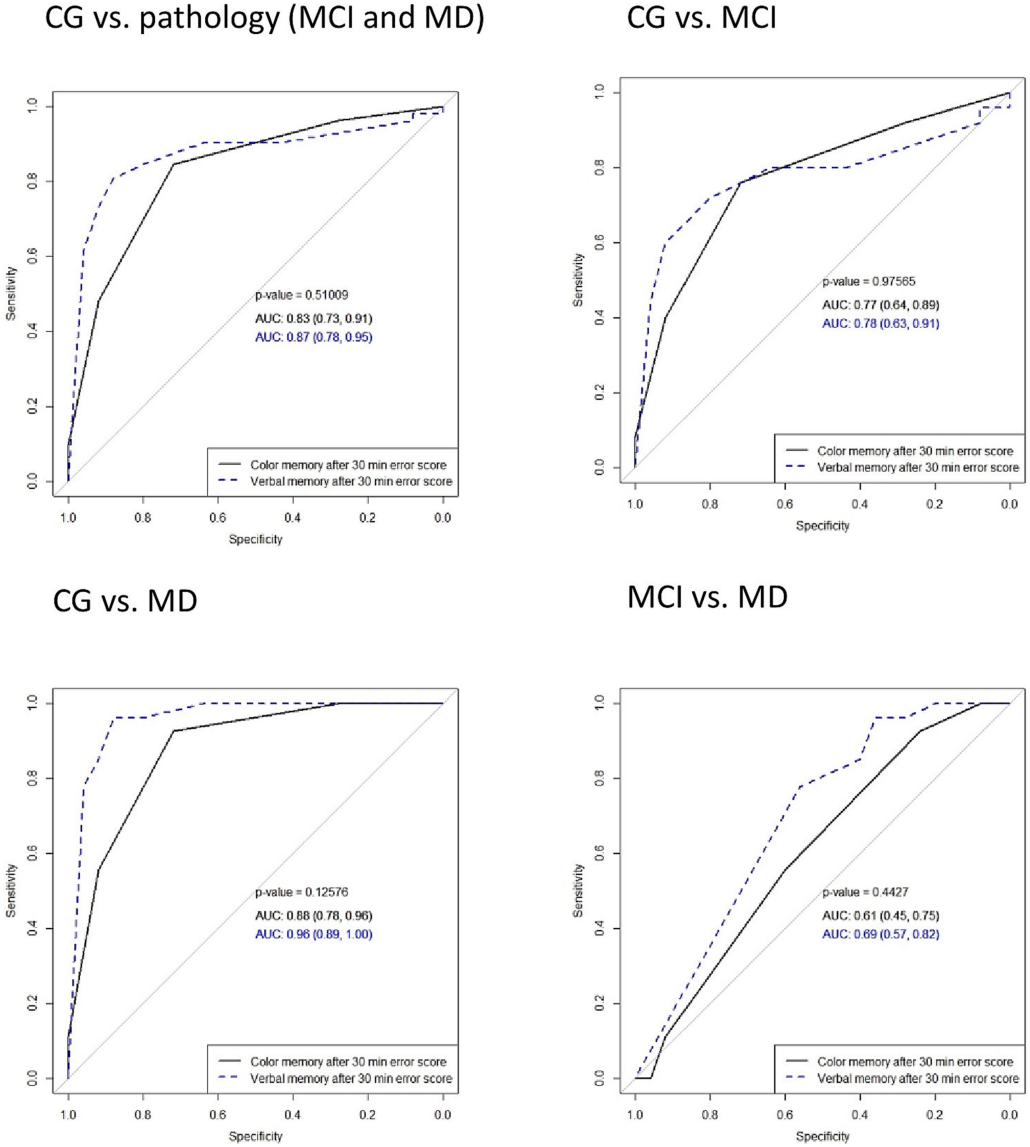
Comparisons of areas under the ROC curves (AUC) in delayed word recall and color recognition after 30 min in verbal and color memory tasks in distinguishing between participant groups. Solid lines represent color memory and dashed lines represent verbal memory test results. MD, mild dementia; MCI, mild cognitive impairment; CG, control group.

**Figure 4:**
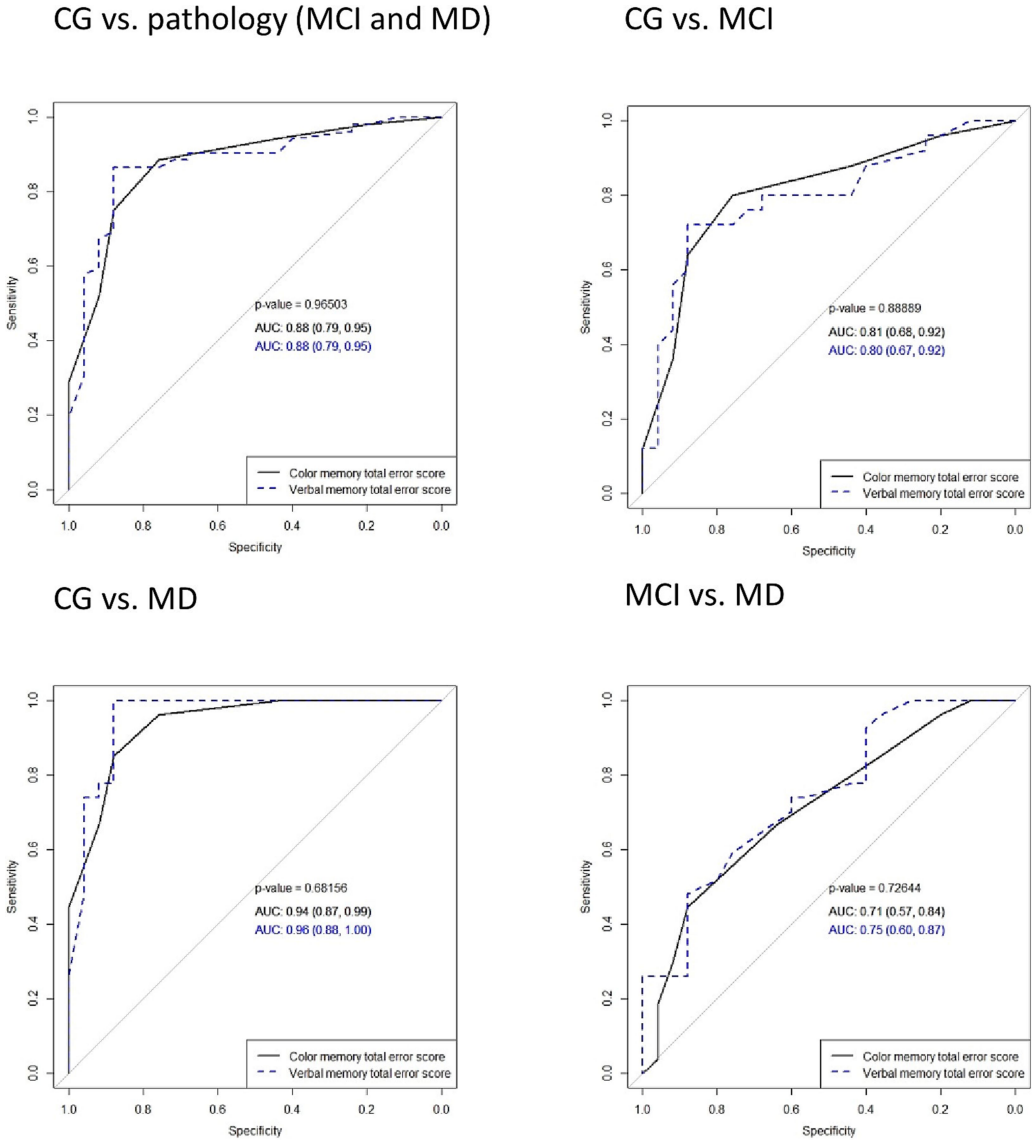
Comparisons of areas under the ROC curves (AUC) in the total error score in color and verbal memory tasks in distinguishing between participant groups. Solid lines represent color memory and dashed lines represent verbal memory test results. MD, mild dementia; MCI, mild cognitive impairment; CG, control group.

To differentiate between CG and AD (MCI or MD) using color and verbal memory error scores, an analysis using the receiver operating characteristic curves (ROC) was conducted. ROC curves of delayed word recall and color recognition after 5 and 30 min, as well as the total error score, showed no significant differences between verbal and color memory tasks in distinguishing the participant groups. The total error score for both tasks demonstrated robust diagnostic properties in differentiating CG from pathology (MCI and MD), with an identical AUC of 0.88. In distinguishing the CG from the MCI, the total error scores in color and verbal memory tasks achieved AUCs of 0.81 and 0.8, respectively. The ROC curves of delayed word recall after 5 min and 30 min, total score of recall (count of words not recalled), delayed color recognition after 5 min and 30 min, and total color memory error score with comparisons among all groups are presented in Figures 2–4.

To distinguish participants with AD (MCI or MD) from CG, a cut-off score of ≥3 errors in total error score of color memory task was established as indicative of AD. A cut-off score of ≥6 errors was established to differentiate patients with MD from those with MCI (with a score of 6 or above considered to be indicative of MD). The diagnostic properties of these thresholds were evaluated and the data are presented in Table 3.

**Table 3:** Diagnostic properties of the total error score color memory task in differentiating between participants with AD and CG.

	CG vs. pathology (MCI and MD), cut-off ≥3 errors	CG vs. MCI, cut-off ≥3 errors	CG vs. MD, cut-off ≥3 errors	MCI vs. MD, cut-off ≥ 6 errors
Sensitivity	88.46% (76.56–95.65%)	80% (59.30–93.17%)	96.3% (81.03–99.91%)	44.44% (25.48–64.67%)
Specificity	76% (54.87–90.64%)	76% (54.87–90.64%)	76% (54.87–90.64%)	88% (68.78–97.45%)
Negative predictive value	76% (59.11–87.40%)	79.17% (62.73–89.56%)	95% (73.27–99.25%)	59.46% (50.4–67.92%)
Positive predictive value	88.46% (79.12–93.94%)	76.92% (61.76–87.31%)	81.25% (68.24–89.73%)	80% (56.07–92.61%)
Overall diagnostic accuracy	84.42% (74.36–91.68%)	78% (64.04–88.47%)	86.54% (74.21–94.41%)	65.38% (50.91–78.03%)

Data are presented as percentage and 95% confidence interval. MD, mild dementia; MCI, mild cognitive impairment; CG, control group.

To examine the association between the independent variables and classification into the three groups, a multinomial logistic regression analysis was conducted.

Initially, a model was developed using age, education, sex, and ADAS-Cog 13 score as predictor variables. Adding predictor variables enhanced the model’s fit to the data, improving upon the initial model that contained only the intercept (*X*^2^ = 85.881, *p* < 0.001; Nagelkerke *R*^2^ = 0.756). The overall percentage of accurately categorized cases using this model was 77.9% (96% CG, 64% MCI, and 74.1% MD cases correctly categorized), with ADAS-Cog 13 score being the strongest and most significant predictor (*X*^2^ = 76.074, *p* < 0.001).

When word recall after 30 min was incorporated into this model, the expanded model’s diagnostic accuracy remained similar, and word recall after 30 min was not a significant predictor (*X*^2^ = 85.86, *p* < 0.001; Nagelkerke *R*^2^ = 0.77). ADAS-Cog 13 score remained a significant predictor (*X*^2^ = 39.29, *p* < 0.001). The overall percentage of cases that were accurately categorized using this model was 75.3% (92% CG, 64% MCI, and 74.1% MD cases).

Furthermore, color recognition after 30 min was added to the initial model, and the expanded model showed a significant improvement in fit over a null model (*X*^2^ = 101.23, *p* < 0.001; Nagelkerke *R*^2^ = 0.823). This model accurately categorized 83.1% of the cases (96% CG, 72% MCI, and 81.5% MD cases were correctly classified). Both ADAS-Cog 13 and color recognition after 30 min were significant predictors, with *X*^2^ values of 62.47 and 15.35, respectively, (*p* < 0.001).

Finally, when the color memory total error scores were added to the initial model, the expanded model (including age, education, sex, ADAS-Cog 13 scores, and color memory total error scores) also showed a significant improvement in fit over a null model (*X*^2^ = 117.176, *p* < 0.001; Nagelkerke *R*^2^ = 0.880). This model accurately categorized 84.4% of the cases (100% CG, 76% MCI, and 77.8% MD cases). Both ADAS-Cog 13 and color memory scores were strong and significant predictors, with *X*^2^ values of 63.88 and 31.30, respectively, (*p* < 0.001).

## 4 Discussion

Our findings revealed that color recognition memory is impaired in the early stages of AD and is more pronounced in the mild dementia stages. Consistent with previous studies, visual perceptual dysfunction is considered an early indication of MCI due to AD and the progression to AD, and the aspects that showed diagnostic potential were perception or memory of figure or structure (7, 25, 26).

In this study, significant differences in color recognition memory were observed between the CG and the MCI group, emphasizing the usefulness of color memory evaluation in the early stages of AD. The color recognition task is a memory task that relies on hippocampus integrity, a region known to be implicated in AD (27). The ventral stream of the visual perception pathway is responsible for color identification and recognition (17). According to recent studies, better visual recognition is associated with the memory of the categorical features of a visual scene aiding in discriminating between targets and lures. During a visual recognition task, activation with categorical representations in the precuneus, inferior temporal cortex, and superior occipital cortex improved scene memory (28). Relying solely on verbal memory tasks to evaluate cognitive function may lead to the overlooking of visual memory deficits. Incorporating visual memory tasks for cognitive evaluations may be useful in improving the diagnostic process. Oltra-Cucarella et al. emphasized that although verbal amnestic MCI was the most prevalent form, a substantial number of participants exhibited either visual-only amnestic MCI or a combination of visual and verbal amnestic MCI. The likelihood of developing AD was equivalent in individuals with visual amnestic or verbal amnestic MCI alone, and higher in those with combined amnestic MCI (29). Squarzoni et al. concluded that visual compared to verbal episodic memory scores correlated most significantly with the presence of amyloid-*β*, as measured by positron emission tomography scans (30). As reported by Cecchini et al., the short-term memory binding test, which evaluates the capacity to retain the integration of surface features such as shapes and colors, has shown sensitivity to indicators of AD pathology and may function as a cognitive marker within the AD continuum (31). Contrary to our study findings, Meyer et al. concluded that episodic recognition memory, based on the incidental learning of visual associations, is largely preserved in comparison with recall in cases of amnestic MCI and mild AD (32, 33). The stimuli utilized in this study comprised 24 line drawings, indicating that our research may provide novel insights into the similarity between color recognition memory and verbal recall memory in early AD, and that color recognition memory may be more impaired than object recognition memory. Our findings underscore the necessity of incorporating both verbal and visual memory evaluations into neuropsychological examinations to identify amnestic MCI with greater precision.

The color recognition task scores demonstrated a significant and strong correlation with the MMSE results, and a moderate correlation with the ADAS-Cog 13 and CDR-SB scores. A linear regression analysis significantly predicted the total error scores for color memory using these three tests. Factors such as age, sex, and education did not have a substantial impact on color memory scores. These results confirm that color memory impairment is associated with the pathological processes of AD rather than other factors known to influence visual perception such as age (7–10). This study also supports the finding that AD-related neuropathological lesions described from the retina to all parts of the visual system affect visual function (7–9).

Recent studies have used color discrimination tasks to differentiate AD from other dementias (18–20). Unger et al. used a color vision task (Farnsworth D-15 color vision test) to distinguish AD from dementia with Lewy bodies, and found that color vision impairment could be utilized as a diagnostic tool to differentiate dementia with Lewy bodies from AD. However, a significant proportion (20%) of patients with AD have color vision impairment. Furthermore, lower cognitive test scores and visuospatial or executive function subscores were associated with color vision impairment in patients with dementia and Lewy bodies, suggesting that this impairment is more closely related to cognitive decline in the later stages of the disease (20). Our study’s findings indicate that the Ishihara color vision test results revealed significant differences between the MD and CG groups. However, no significant differences were detected between the CG and MCI groups or between the MCI and MD groups. These results suggest that alterations in color vision may be attributed to progressive neurodegeneration (34).

Research on the diagnostic potential of color memory tests remains limited because evaluations of color vision and memory are infrequent. This study revealed that the color recognition memory task effectively distinguished participants with early AD from cognitively normal older adults (AUC = 0.88) and those with prodromal AD from cognitively normal older adults (AUC = 0.81). Additionally, the incorporation of color memory scores into multinomial logistic regression models resulted in a notable improvement in overall classification accuracy. This enhancement led to an increase in the three-group classification accuracy from 77.9 to 84.4%, representing a 6.5% improvement. Notably, the precise identification of MCI showed considerable improvement, increasing from 64 to 76%, representing a 12% enhancement in precision. Identifying MCI is particularly difficult in primary care settings. According to the 2024 World Alzheimer Report (4, 6), approximately 80% of the population, and more concerningly, 65% of medical professionals erroneously perceive dementia as a normal part of aging. This misconception can delay early detection and timely access to necessary treatment and assistance (5). Color memory testing may be beneficial in enhancing diagnostic accuracy, particularly when distinguishing between MCI due to AD from age-related changes.

Although the color memory task showed satisfactory diagnostic performance in differentiating participants with early AD from older adults with normal cognition, its ability to differentiate among MCI and mild dementia due to AD was suboptimal. One explanation for these results is that color memory impairment most likely occurs early in the disease course. These changes are evident even in the early stages of AD, thereby reducing their effectiveness in tracking disease progression. This finding aligns with that of Ritchie et al., who found that middle-aged individuals whose parents had been diagnosed with dementia showed lower than expected scores on visual memory tests, whereas their performance in verbal memory assessments remained normal (35).

This study has a few limitations. Firstly, our results were limited by the cross-sectional study design; future longitudinal studies are needed to assess the progression of color memory impairment during the course of AD. Secondly, cerebrospinal fluid biomarker analysis was conducted for a small proportion of participants with MCI and MD, and positron emission tomography scans were not performed. Incorporating these methods would have offered valuable insights into how changes in color memory relate to the accumulation of brain Aβ and the deterioration of neurons. Thirdly, the appearance of colors on a computer screen usually differs across devices due to variation in display settings. To minimize the effects of this limitation, we used distinct colors, each varying by approximately 25% in the red, blue, or green components of the RGB scale. It is well established that ocular conditions such as glaucoma, diabetic retinopathy, and macular degeneration can impact color vision even in their initial stages (36–39). Although all participants in this study had documented prior eye examinations, a recent ophthalmological assessment was not conducted. Consequently, it is not possible to ascertain whether the included patients had ophthalmological conditions that could introduce a selection bias at the time of recruitment. Finally, despite the observed significant differences, the small sample size may limit the generalizability of our findings. A larger sample size is necessary to confirm our findings.

In conclusion, the test results for color recognition memory showed significant differences between older adults with normal cognition and those with early AD, highlighting its potential in the early diagnosis of AD. Color memory testing has good diagnostic characteristics and can be an easy-to-execute, reliable, and noninvasive diagnostic test for early AD.

## Data Availability

The raw data supporting the conclusions of this article will be made available by the authors, without undue reservation.
