# Supplementary material for: Color memory as a diagnostic test for mild cognitive impairment and early stage of Alzheimer’s disease
Source: Front Neurol. 2025 May 27;16:1589335. doi: 10.3389/fneur.2025.1589335 (PMC12148855; doi:10.3389/fneur.2025.1589335)
Supplement: Supplementary file 1 [file Table_1.docx]

Supplementary Material

## Supplementary Table

|  | Green | Blue | Red | Black | White | Grey | Orange | Violet | Pink | Brown | Light blue | Yellow |
| --- | --- | --- | --- | --- | --- | --- | --- | --- | --- | --- | --- | --- |
| CG | 0 | 6 | 0 | 0 | 6 | 5 | 4 | 5 | 4 | 4 | 9 | 2 |
| MCI | 7 | 9 | 9 | 2 | 13 | 7 | 9 | 8 | 11 | 4 | 8 | 6 |
| MD | 10 | 13 | 10 | 9 | 13 | 14 | 12 | 13 | 9 | 11 | 13 | 12 |

**Supplementary Table 1.** Total error score for each color in three groups. MD, mild dementia; MCI, mild cognitive impairment; CG, control group.
